# Testing of candidate single nucleotide variants associated with paclitaxel neuropathy in the trial NCCTG N08C1 (Alliance)

**DOI:** 10.1002/cam4.625

**Published:** 2016-01-14

**Authors:** Ganesh K. Boora, Rahul Kanwar, Amit A. Kulkarni, Alexej Abyzov, Jeff Sloan, Kathryn J. Ruddy, Michaela S. Banck, Charles L. Loprinzi, Andreas S. Beutler

**Affiliations:** ^1^ Department of Medical OncologyMayo ClinicRochesterMinnesota; ^2^ Department of Health Sciences Research (Biostatistics and Informatics)Mayo ClinicRochesterMinnesota; ^3^ Alliance Statistics and Data CenterMayo ClinicRochesterMinnesota; ^4^ Mayo Clinic Cancer CenterRochesterMinnesota

**Keywords:** Chemotherapy‐induced peripheral neuropathy, CIPN, genetics

## Abstract

Paclitaxel‐induced peripheral neuropathy (PIPN) cannot be predicted from clinical parameters and might have a pharmacogenomic basis. Previous studies identified single nucleotide variants (SNV) associated with PIPN. However, only a subset of findings has been confirmed to date in more than one study, suggesting a need for further re‐testing and validation in additional clinical cohorts. Candidate PIPN‐associated SNVs were identified from the literature. SNVs were retested in 119 patients selected by extreme phenotyping from 269 in NCCTG N08C1 (Alliance) as previously reported. SNV genotyping was performed by a combination of short‐read sequencing analysis and Taqman PCR. These 22 candidate PIPN SNVs were genotyped. Two of these, rs7349683 in the *EPHA5* and rs3213619 in *ABCB1* were found to be significantly associated with PIPN with an Odds ratios OR = 2.07 (*P* = 0.02) and OR = 0.12 (*P* = 0.03), respectively. In addition, three SNVs showed a trend toward a risk‐ or protective effect that was consistent with previous reports. The rs10509681 and rs11572080 in the gene CYP2C8*3 showed risk effect with an OR = 1.49 and rs1056836 in *CYP1B1* showed a protective effect with an OR = 0.66. None of the other results supported the previously reported associations, including some SNVs displaying an opposite direction of effect from previous reports, including rs1058930 in CYP2C8, rs17222723 and rs8187710 in *ABCC2*, rs10771973 in *FGD4*, rs16916932 in CACNB2 and rs16948748 in *PITPNA*. Alliance N08C1 validated or supported a minority of previously reported SNV‐PIPN associations. Associations previously reported by multiple studies appeared to have a higher likelihood to be validated by Alliance N08C1.

## Introduction

Chemotherapy‐induced peripheral neuropathy (CIPN) is a toxicity that adversely affects a substantial minority of patients treated with paclitaxel, while others receiving the same drug remain unaffected 1. Paclitaxel‐induced peripheral neuropathy (PIPN) cannot be predicted for individual patients from clinical parameters. Therefore, a pharmacogenomic basis has been suggested to explain phenotypic variability in PIPN 2. Early studies on the pharmacogenomics of PIPN tested single nucleotide variants (SNV) in known drug metabolism genes 2. More recent investigations performed genome wide association studies (GWAS) 3. Taken together these studies reported over 20 SNVs to be significantly associated with PIPN or as strong candidates awaiting validation.

Of the reported SNV, only a subset has been included in more than one study and of these only a few have been validated across multiple patient cohorts. An association of rs10509681 in the drug metabolism gene CYP2C8*3 was reported and validated by two independent groups in three different patient cohorts 4, 5, 6, 7.The GWAS study by Baldwin et al. 3 which was based on the clinical trial CALGB 40101, nominated the SNV rs7001034 and rs7833751 in *FZD3*, rs7349683 in *EPHA5*, rs4737264 in *XKR,* and rs10771973 in *FGD4* as candidate PIPN biomarkers 3. Recently, Garcia et al. published a smaller GWAS, which was based on 144 European patients 8, validating two of the SNVs reported by Baldwin et al. (above), rs7349683 (*EPHA5)* and rs4737264 (*XKR*).

However, several associations of SNV with PIPN, including some of those introduced above, could not be confirmed by other studies. Rizzo et al. and Ofverholm et al. failed to confirm an association of rs10509681 (CYP2C8*3) with PIPN 9, 10. The SNV rs1045642 and rs2032582 (*ABCB1*) were each proposed by one report and subsequently refuted in follow‐up studies by others 4, 10, 11, 12, 13.

Recently, Abraham et al. reported novel PIPN risk SNV rs3213619 (*ABCB1*) along with several other candidates 14. The study by Garcia et al. 8 proposed an additional SNV, rs4141404 in *LIMK2* as a candidate biomarker of PIPN, which did not reach the threshold for genome‐wide significance in their report suggesting a need for future independent validation.

Taken together, these reports emphasize the need—as in other fields of genetics—to seek retesting and validation of proposed genetic associations of SNV with PIPN in additional patient cohorts. Recently, we reported a study on the natural history of PIPN based on clinical trial North Central Cancer Treatment Group (NCCTG) N08C1 15 and subsequently used this patient cohort to test the role of Charcot‐Marie‐Tooth disease (CMT) genes for PIPN 16. NCCTG is now part of the Alliance for Clinical Trials in Oncology. Here, we use the same cohort to retest SNVs that were previously reported by others to be associated with PIPN.

## Patients and Methods

### Identification of SNV from previously published reports

A systematic review of the literature was performed to identify previously reported studies in PIPN pharmacogenomics. A MEDLINE/PubMed search with the key words “Chemotherapy induced peripheral neuropathy,” or “paclitaxel neuropathy,” or “taxane pharmacogenetics,” or “paclitaxel neurotoxicity,” was carried out in October 2014. The bibliographies of the identified publications were also reviewed for additional studies. Only those studies that tested direct associations between SNV and PIPN as one of their endpoints were included. Studies that investigated SNV associations with other outcomes were excluded. Only reports in the English language published in peer‐reviewed journals were considered. In the case of publications reporting data on multiple SNV, only positive results were selected for retesting based on the strength of the association reported or its location in a gene with strong evidence for an association with PIPN. All SNV fulfilling the above criteria that were also located in transcribed regions of a gene, that is, exonic or adjacent untranslated regions (UTR) were included in the present study. In addition, 9 intronic SNVs were selected (all from Baldwin et al. 3) because of their prominent role as being identified in the first large GWAS study in the PIPN field. In total, 22 SNVs from 16 genes were selected for testing in the current study.

### Patient selection and phenotyping

The NCCTG (Alliance) N08C1 is a previously reported prospective PIPN clinical trial that was designed to study the natural history of paclitaxel neuropathy 15, 16, 17 and to test correlative genetics. The study cohort was the same as described in our previous report on CMT disease gene sequencing 16. In brief, 269 patients exposed to paclitaxel chemotherapy were evaluated for PIPN by serial repeat assessments with the CIPN20 instrument. Based on the scores from serial assessments using a Rasch type statistical model 18, we estimated a slope representing the rate of PIPN symptom progression. An “extreme phenotyping” approach was used to select “cases” and “controls” from the tails of the neuropathy phenotype distribution, excluding patients with equivocal progression (“uncategorized”) of PIPN symptoms. Extreme phenotyping has been shown to improve the power of genetic association studies as discussed by others 19, 20. This approach identified 119 patients, 73 PIPN cases and 46 controls. Both groups were balanced in terms of demographic and clinical data, including potential confounding factors like age, ethnicity, and diabetes status. Informed consent was obtained from patients for CIPN assessment and collection of blood for genetic testing (Mayo IRB# 08‐006970; 09‐002454).

### Genotyping of candidate SNV

Genomic DNA (gDNA) was isolated from peripheral leukocytes from each patient. Quality control of the DNA was done with Qubit® (ThermoScientific, Wilmington, DE) fluorometer and NanoDrop® (Life Technologies, Green Island, NY) spectrophotometer. Thirteen SNVs in 8 genes were genotyped by the sequencing‐based approach described previously 16. Nine SNVs in 8 genes rs7833751 (*FZD3*), rs7001034 (*FZD3*), rs2233335 (*NDRG1*), rs10771973 (*FGD4*), rs16948748 (*PITPNA*), rs17781082 (*CAND1*), rs16916932 (*CACNB2*), rs4737264 (*XKR4*), rs1903216 (*BCL6*) were genotyped by TaqMan® PCR. PCR amplification of gDNA encompassing the loci of interest was performed to generate DNA amplicons. TaqMan probes directed to the allele at the test loci were designed. Analysis of the fluorescence signal from the Taqman PCR reaction and allele discrimination was performed with the SDS 2.0 software (Applied Biosystems, Waltham, MA). gDNA for Taqman PCR genotyping was available for 114 (of 119) patients (71 cases and 43 controls). Experiments were performed at Mayo Clinic, Rochester, MN.

### Statistical analysis of genotyping results

Statistical analysis was done under the three models of inheritance, additive (primary analysis), dominant, and recessive. Odds ratios (OR) were computed with their corresponding 95% confidence intervals (CIs) using the statistical programming package *R*. Fisher exact test 21was used to compute the type I error rate. A *P*‐value of <0.05 was considered statistically significant. Collection of the clinical data was conducted by the Alliance Statistics and Data Center. All analyses were based on the study database frozen on 17 December 2012.

## Results

**Figure 1:**
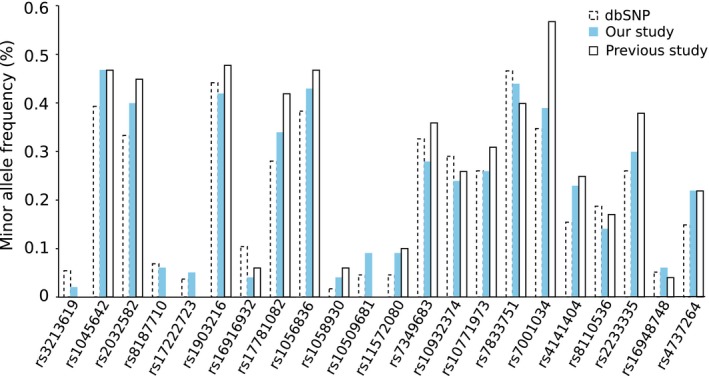
Minor allele frequencies (MAFs) of single nucleotide variants (SNV). A Graphical representation of MAFs of the tested SNV in dbSNP database, present study (N08C1) and previous study reported (MAF taken from previous study where data was available) shows that they were similar.

The 22 SNVs in 16 genes were genotyped. The minor allele frequencies (MAFs) in the present study were similar to the original report or, in cases of divergence, tended to be in‐between the original report and the MAF found in the reference dataset dbSNP (Fig. 1).

**Figure 2:**
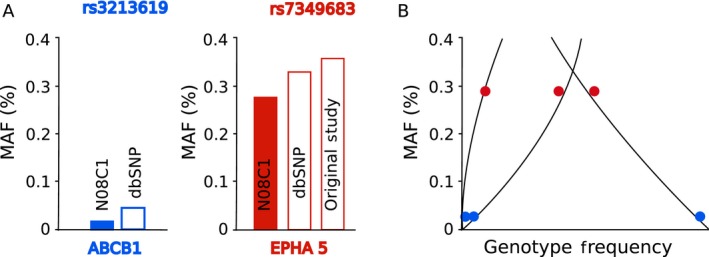
Quality control of genotyping of the two significant single nucleotide variants (SNV). (A) The observed minor allele frequency of the two SNV in ABCB1 and EPHA5 is shown in solid color bars. The observed MAF in ABCB1 is similar to dbSNP (MAF from prior study not available) and in case of EPHA5 it is similar to dbSNP and prior study. (B) The allele frequencies of both the SNV (homozygous major, minor, and heterozygous) are in agreement with Hardy–Weinberg equilibrium.

**Figure 3:**
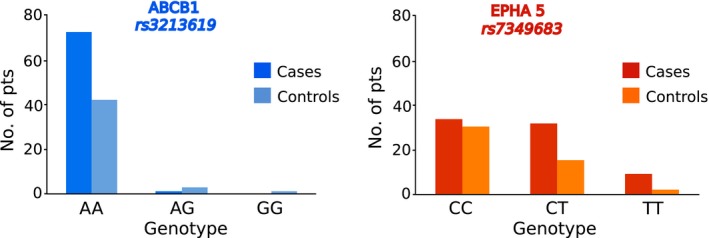
Genotype distribution of single nucleotide variants (SNV) in EPHA5 and ABCB1 between cases and controls: Number of cases and controls harboring the three possible genotypes (homozygous major, homozygous minor and heterozygous) is shown here.

Two SNVs were found to be significantly associated with PIPN, rs7349683 in the gene *EPHA5* and rs3213619 in the gene ABCB1. The MAFs of the two SNVs were found to be similar as in the reference dataset dbSNP (Fig. 2A). Genotype frequencies were in agreement with Hardy–Weinberg equilibrium (Fig. 2B). Allele counts are provided in Figure 3. For rs7349683 in EPHA5, the variant was significantly associated with PIPN with an OR = 2.07 (*P* = 0.02) and the same direction of effect as the original study 3. Here the rs3213619 in *ABCB1* had an OR = 0.12 (*P* = 0.03) with the minor allele being protective, as in the original report 14.

Of the remaining SNVs, some showed a trend toward a risk or protective effect in the same direction as the original report without meeting the cutoff for statistical significance (of *P* < 0.05 in the present study). The two SNVs rs10509681 and rs11572080 in the gene CYP2C8*3, which are in perfect linkage disequilibrium (LD) (*R* ^2^ = 1), showed a PIPN risk phenotype with an OR = 1.49 for the additive model and OR = 1.56 for the dominant model in the present study. This compared with previous studies having reported a range of findings, which included strong effects with an OR = 3.13 (dominant model reported only) 5, a HR = 1.93 (additive model reported only) 7, and a HR = 1.72 (additive model reported only) 6, and three other studies that had presumably low effect sizes (not specified by the respective report) because they reported no association of CYP2C8*3 SNV with PIPN 9, 11, 22. The SNV rs1056836 in *CYP1B1* showed a protective effect with an OR = 0.66 in the present study, which was a similar effect size and in the same direction as the original report 14.

The remaining SNV had an OR of 0.67–1.5, considered here to be a negligible effect (close to OR = 1), or had an opposite direction of effect from the original report. Eleven SNVs had an OR closer to 1 which include rs7001034 in *FGD3* had an OR = 0.9 compared with OR = 0.57 in the previous report 3. The following variants trended in the opposite direction in the present cohort compared with previous studies: the variant rs1058930 in *CYP2C8* had an OR = 0.26 (*P* = 0.05) in contrast to the original study by Abraham et al. that had a reported risk effect with an OR = 1.48 14. rs16916932 in *CACNB2* had an OR = 0.29 (*P* = 0.08) in the present cohort, while an OR = 2.08 had been observed in the prior study 3. The SNV rs17222723 and rs8187710 in gene *ABCC2* that are in strong LD had an OR = 1.94 and 1.97, respectively, in contrast to the previously published reports that showed a decreased risk of PIPN with an OR = 0.66 and OR = 0.63 14. The SNV rs10771973 in *FGD4* had an OR = 0.56 and rs16948748 in *PITPNA* had an OR = 0.60 also trending in the opposite direction of the previous report, where the HR was 1.57 and 2.37, respectively 3.

The 11 SNVs that were contradicting or not supportive of prior reports and considered here as negative had MAF in the present study that were similar (within 1.5‐fold) to the original report and to reference datasets, and none diverged significantly from the allele distribution expected on the basis of a Hardy–Weinberg equilibrium. For the SNV rs1045642 and rs2032582 (*ABCB1*), rs1903216 (*BCL6*), rs2233335 (*NDRG1*), rs7001034 (*FZD3*), and rs4737264 (*XKR4*), the CI around the OR in the present study encompassed the OR in the original report, suggesting that the power of the present study was not sufficient to draw conclusions. For the rs17781082 (CAND1), and rs7833751 (FZD3) the previously reported OR was outside of the CI for the present study, suggesting that the new cohort provided contradicting evidence. (For rs10932374 (ERBB4), rs4141404 (LIMK2), and rs8110536 (MISP/C19orf21) HR instead of OR were provided in the original studies.)

## Discussion

PIPN is a clinically highly relevant complication of chemotherapy with paclitaxel, a widely used cancer treatment. To date several PIPN pharmacogenomic studies have been reported. A review of this literature suggested that only a few SNV‐PIPN associations were supported by multiple studies and that most current SNV candidates were either supported and then contradicted in different cohorts or had been reported only in a single study. This overall limited agreement between different reports in terms of SNV‐PIPN associations may be related to multiple reasons, including different study designs such as the use of different PIPN phenotyping methods, or could be the result of publication bias 23 or under‐correcting of multiple testing in studies, using high‐throughput genetic discovery methods.

N08C1 is a recently reported new PIPN cohort that differs from the previously performed PIPN pharmacogenomics studies by its extensive serial PIPN phenotyping with the CIPN20 instrument. N08C1 is therefore a suitable cohort for attempting to validate previous observations from other studies. N08C1 represents a relatively small cohort of only 119 patients. The limited cohort size is in part due to the rigorous patient selection criteria and extreme phenotyping approach used; these factors at the same time mitigate the impact of limited study size by using the patients that can provide the strongest signal. Study size impacts the confidence of the results differently for each SNV depending on its MAF. We addressed this by reporting the statistical CI for each OR in Table 1 and taking it into account when assessing the findings. Retesting of the previously reported SNV‐PIPN associations in the present study demonstrated a moderate agreement. N08C1 successfully replicated the association of the two SNV in *EPHA5* and *ABCB1* with PIPN. Thereby, N08C1 is the third study supporting rs7349683 in *EPHA5* as a PIPN biomarker following its original nomination by Baldwin et al. (HR = 1.63; 95% CI 1.34–1.98; *P *= 9.6 × 10^−7^) 3 and subsequent validation by Garcia et al. (HR = 1.68; *P *= 1.4 × 10^−9^ on meta‐analysis) 8. Both these studies used National Cancer Institute Common Toxicity Criteria for Adverse Events (NCI‐CTCAE) for phenotyping. The second significant SNV is rs3213619 in *ABCB1*. This SNV was significantly associated with reduction of risk of PIPN in the study by Abraham et al. which reported an OR = 0.47 (95% CI 0.28–0.79, *P = *0.004) in a study of 1303 patients. The present study replicated the finding (*P* = 0.03) 14. In addition to the above two, the SNV in CYP2C8*3 was associated with risk of neuropathy without reaching statistical significance. The association of variant in CYP2C8*3 was first reported by Green et al. 4 and later validated by Hertz et al. 5, 6, 7. Thus, the SNV validated in the present report tended to be among those for which multiple studies had lent support previously. On the other hand, all of those SNVs for which the present study found opposite effects, that is, strongly contradicted, were put forth only in a single previous study.

**Table 1:** Results of the 22 SNV genotyped

Gene	SNV							Study	Additive					Dominant					Recessive				
	rsID	ref	alt	chr	Pos (GRCh37)	MAF	HWE		OR	HR	*P*‐value	95% CI		OR	HR	*P*‐value	95% CI		OR	HR	*P*‐value	95% CI	
ABCB1	rs1045642	G	A	7	87509329	0.48	0.58	N08C1	1.08		0.79	0.62	1.88	1.37		0.53	0.56	3.32	0.86		0.83	0.34	2.24
								Abraham 14	0.83		0.03												
	rs2032582	C	A	7	87531302	0.40	0.70	N08C1	0.97		1.00	0.54	1.73	0.99		1.00	0.42	2.31	0.92		1.00	0.31	2.85
								Abraham 14	1.22		0.02												
	rs3213619	A	G	7	87600877	0.03	0.06	N08C1	0.12		0.03	0.00	1.11	0.15		0.07	0.00	1.56	0.00		0.39	0.00	24.5
								Abraham 14	0.47		0.004												
ABCC2	rs17222723	T	A	10	99836239	0.05	1.00	N08C1	1.94		0.38	0.47	11.46	2.00		0.37	0.47	12.16	0.00		1.00	0.00	∞
								Abraham 14	0.66		0.05												
	rs8187710	G	A	10	99851537	0.07	1.00	N08C1	1.97		0.30	0.57	8.63	2.05		0.28	0.57	9.34	0.00		1.00	0.00	∞
								Abraham 14	0.63		0.02												
BCL6	rs1903216	G	A	3	187911715	0.42	0.44	N08C1	0.91		0.78	0.51	1.63	0.93		1.00	0.39	2.19	0.85		0.81	0.30	2.5
								Abraham 14	1.59		5.6 × 10^−6^												
CACNB2	rs16916932	C	T	10	18187347	0.04	1.00	N08C1	0.29		0.08	0.05	1.39	0.27		0.08	0.04	1.36	0.00		1.00	0.00	∞
								Baldwin 3	2.08		4.3 × 10^−6^												
CAND1	rs17781082	C	T	12	67082547	0.34	0.41	N08C1	0.82		0.56	0.45	1.51	0.74		0.56	0.32	1.71	0.87		0.78	0.25	3.2
								Baldwin 3	1.6		4.3 × 10^−6^												
CYP1B1	rs1056836	G	C	2	38071060	0.44	0.02	N08C1	0.66		0.14	0.38	1.15	0.63		0.33	0.26	1.46	0.59		0.27	0.23	1.51
								Abraham 14	0.81		0.02												
CYP2C8	rs10509681	T	C	10	95038992	0.10	0.60	N08C1	1.49		0.50	0.55	4.48	1.56		0.48	0.54	4.92	0.00		1.00	0.00	∞
								Hertz 5						3.13		0.07							
								Hertz 6		1.72	0.018												
								Hertz 7		1.93	0.032												
	rs11572080	C	T	10	95067273	0.10	0.60	N08C1	1.49		0.50	0.55	4.48	1.56		0.48	0.54	4.92	0.00		1.00	0.00	∞
								Leskela 12		1.72	0.032												
								Hertz 5						3.13		0.075							
								Hertz 6		1.72	0.018												
	rs1058930	G	C	10	95058362	0.04	1.00	N08C1	0.26		0.05	0.04	1.16	0.24		0.04	0.04	1.13	0.00		1.00	0.00	∞
								Abraham 14	1.48		0.04												
EPHA5	rs7349683	C	T	4	65332086	0.28	0.50	N08C1	2.07		0.03	1.08	4.10	2.26		0.04	1.00	5.26	3.07		0.20	0.59	30.5
								Baldwin 3	1.63		9.6 × 10^−7^												
								Garcial 8		1.68	1.4 × 10^−9^												
ERBB4	rs10932374	G	A	2	211379678	0.24	0.13	N08C1	0.75		0.36	0.39	1.45	0.66		0.34	0.29	1.50	0.94		1.00	0.21	4.81
								Garcia 8		2.25	2.58 × 10^−5^												
FGD4	rs10771973	G	A	12	32640040	0.25	0.22	N08C1	0.56		0.06	0.29	1.07	0.55		0.17	0.24	1.28	0.37		0.17	0.07	1.68
								Baldwin 3	1.57		2.6 × 10^−6^												
FZD3	rs7001034	G	A	8	28505861	0.39	0.55	N08C1	0.90		0.78	0.50	1.63	0.87		0.84	0.35	2.07	0.87		0.78	0.25	3.24
								Baldwin 3	0.57		3.1 × 10^−9^												
	rs7833751	G	T	8	28505275	0.45	0.35	N08C1	1.30		0.41	0.73	2.32	1.42		0.52	0.56	3.53	1.51		0.61	0.49	5.24
								Baldwin 3	0.58		7.5 × 10^−9^												
LIMK2	rs4141404	C	A	22	31279199	0.24	0.31	N08C1	1.07		0.88	0.55	2.09	1.17		0.71	0.52	2.65	0.62		0.64	0.04	8.87
								Garcia 8		2.41	3.22 × 10^−6^												
MISP/	rs8110536	T	G	19	756985	0.14	0.25	N08C1	0.77		0.57	0.35	1.73	0.64		0.39	0.26	1.63	1.92		1.00	0.15	103.4
C19orf21								Garcia 8		2.24	2.98 × 10^−5^												
NDRG1	rs2233335	T	G	8	133248822	0.31	1.00	N08C1	1.06		0.88	0.57	2.00	0.99		1.00	0.43	2.27	1.42		0.74	0.30	8.99
								Baldwin 3	0.65		5.2 × 10^−5^												
PITPNA	rs16948748	T	G	17	1554545	0.06	1.00	N08C1	0.60		0.40	0.17	2.10	0.58		0.39	0.16	2.12	0.00		1.00	0.00	∞
								Baldwin 3	2.37		2.7 × 10^−6^												
XKR4	rs4737264	A	C	8	55198762	0.23	1.00	N08C1	0.86		0.74	0.44	1.72	0.78		0.56	0.34	1.80	1.22		1.00	0.17	14.08
								Baldwin 3	1.68		1.9 × 10^−6^												
								Garcia 8		1.71	3.1 × 10^−8^												

MAF, minor allele frequency; HWE, Hardy–Weinberg equilibrium *P*‐value.

The SNVs validated in the present study are found in genes involved in peripheral nervous system biology or in drug metabolism. The gene *EPHA5* belongs to the ephrin family of tyrosine kinase receptors involved in communication and signaling between different cell types in the nervous system 24. *EPHA5* signaling plays an important role in early stages of synaptogenesis 25. *ABCB1,* also known as *MDR1* (multidrug resistant gene), is a part of the ABC transporter superfamily. It encodes a P‐glycoprotein (Pgp), which was first described in multi‐drug resistant cells 26. It is involved in absorption, disposition, and metabolism of drugs 27. The genes *CYP2C8* and *CYP3A4* are involved in the metabolism of paclitaxel. Variants in gene *CYP2C8* decrease paclitaxel metabolic activity and lead to increased drug exposure 28, 29.

Taken together, these results indicate that SNV associations with CIPN should be supported by multiple independent studies before any consideration as a clinical biomarker.

## Conflict of Interest

The authors declare no conflict of interest.
